# Exploring the Cross-cultural Acceptability of Digital Tools for Pain Self-reporting: Qualitative Study

**DOI:** 10.2196/42177

**Published:** 2023-02-08

**Authors:** Syed Mustafa Ali, Rebecca R Lee, John McBeth, Ben James, Sean McAlister, Alessandro Chiarotto, William G Dixon, Sabine N van der Veer

**Affiliations:** 1 Centre for Epidemiology Versus Arthritis, Division of Musculoskeletal and Dermatological Sciences, Manchester Academic Health Science Centre The University of Manchester Manchester United Kingdom; 2 Centre for Health Informatics, Division of Informatics, Imaging and Data Science, Manchester Academic Health Science Centre The University of Manchester Manchester United Kingdom; 3 uMotif London United Kingdom; 4 Department of General Practice, Erasmus University Medical Center Rotterdam Netherlands

**Keywords:** chronic pain, pain perception, cross-cultural comparison, pain measurement, mobile app, mobile phone

## Abstract

**Background:**

Culture and ethnicity influence how people communicate about their pain. This makes it challenging to develop pain self-report tools that are acceptable across ethnic groups.

**Objective:**

We aimed to inform the development of cross-culturally acceptable digital pain self-report tools by better understanding the similarities and differences between ethnic groups in pain experiences and self-reporting needs.

**Methods:**

Three web-based workshops consisting of a focus group and a user requirement exercise with people who self-identified as being of Black African (n=6), South Asian (n=10), or White British (n=7) ethnicity were conducted.

**Results:**

Across ethnic groups, participants shared similar lived experiences and challenges in communicating their pain to health care professionals. However, there were differences in beliefs about the causes of pain, attitudes toward pain medication, and experiences of how stigma and gender norms influenced pain-reporting behavior. Despite these differences, they agreed on important aspects for pain self-report, but participants from non-White backgrounds had additional language requirements such as culturally appropriate pain terminologies to reduce self-reporting barriers.

**Conclusions:**

To improve the cross-cultural acceptability and equity of digital pain self-report tools, future developments should address the differences among ethnic groups on pain perceptions and beliefs, factors influencing pain reporting behavior, and language requirements.

## Introduction

### Pain Inequalities

Chronic pain affects approximately 28 million people in the United Kingdom alone [1], causing both personal and economic burden [2]. To reduce this burden, it is essential to accurately measure pain, know its causes, and estimate its impact on people’s lives [3]. There are inequalities in pain prevalence, pain intensity, and pain treatment that have been linked to people’s characteristics, including their socioeconomic status, geographical location, and ethnicity [4,5]. For example, lower socioeconomic status is associated with higher bodily pain levels in the United Kingdom, Australia, and Germany, particularly in older people [6], and pain is more prevalent among the Black and Asian ethnic minorities [7]. Asians are less likely to receive pain medication than White patients [8], and Black individuals may have different pain management preferences and expectations [9].

### Influence of Culture and Ethnic Background on Pain Experience

Inequalities in pain may be partly explained by the influence of culture and ethnicity on pain perception and reporting. A person’s cultural and ethnic background may affect the way he/she perceives, experiences, and communicates pain [10], and people from different ethnic groups tend to give different meanings to pain [11]. In turn, these inequalities may impact the quality and content of patient-provider communication on pain [12,13].

The influence of the cultural and ethnic background on an individual’s pain experiences and reporting behaviors makes it challenging to develop tools for self-reporting pain that are acceptable and valid across ethnic groups. For example, a review of the cultural adaptations of the Pain Catastrophizing Scale found that construct (ie, varying correlation with other pain scores) and structural (ie, differences in subscales) validity varied across translated versions [14]. Moreover, a review by Booker and Herr [15] found that many pain assessment tools lacked evidence of their validity and reliability in ethnically diverse populations. Another review reported that digital pain self-report and self-management apps seldom offered culturally tailored aspects [16], potentially hampering their cross-cultural acceptability. Similarly, a review of smartphone-based pain manikins found that the manikin appearance could seldom be culturally personalized [17].

### Objectives of This Study

The aim of this study was to inform the design and development of cross-culturally acceptable digital pain self-report tools by better understanding individuals’ pain experiences and reporting behaviors across ethnic backgrounds. The specific objectives were to explore similarities and differences across ethnic groups in (1) the description of pain experience and its reporting and (2) user requirements for digital pain self-report tools by using a smartphone-based pain manikin as an example. We expect this to contribute to acceptable and, ultimately, valid digital pain self-report for people living with a painful condition, irrespective of their ethnic backgrounds.

## Methods

### Study Design

We conducted 3 web-based workshops, each consisting of a focus group discussion and a user requirement exercise. The focus group discussions addressed the first objective. This phenomenological approach acknowledges and explores the subjective experience, which can be used to develop or reorient our understanding of the phenomenon under consideration [18]. We explored the phenomenon of pain experience, its reporting, and how it is embedded within individuals’ cultural and ethnic backgrounds. We used the consolidated criteria for reporting qualitative research checklist to guide reporting of this part of our study [19]. For the second objective, we analyzed user requirements by using the Table of Specifications approach [20] to guide discussions on important aspects of digital pain self-report tools by using a smartphone-based manikin as an example. This approach attempts to translate a set of concepts (in our case, aspects of pain experience and reporting) into a set of items that can be used to assess them.

### Ethics Approval

The study received a favorable opinion and Health Research Authority approval from the National Health Services Westminster Research Ethics Committee (ref 21/PR/0342).

### Eligibility and Recruitment of Participants

Adults (older than 18 years) were eligible to take part in this study if they lived in the United Kingdom and self-identified as (1) living with a primary (ie, pain without any underlying condition) or secondary pain condition (eg, ankylosing spondylitis, rheumatoid arthritis) for more than 3 months and (2) being Pakistani or Bangladeshi, Black African, or White British. Using a purposive sampling approach, we invited people of specific ethnicities who had participated in a related study on the feasibility of a pain self-reporting tool using a smartphone-based pain manikin (Ali SM et al, unpublished data, January 2023). We also recruited potential participants via online community groups (eg, WhatsApp groups for Black Africans, a Facebook group for Pakistanis), as well as online groups of people with an interest to take part in research studies through convenient sampling. We shared a study flyer (Multimedia Appendix 1) with them, after which people could express their interest in taking part. One researcher (SMA) then determined people’s eligibility by telephone screening and asked those eligible to provide informed written consent via email.

### Data Collection

We organized 3 web-based ethnicity-specific workshops consisting of focus groups followed by a user requirement exercise on Zoom: one with South Asians, one with Black Africans, and one with White British. All workshops had the same topic guide (Multimedia Appendix 2), and each was scheduled to last for 2 hours. Before the workshops, participants completed a web-based questionnaire, capturing key demographics (age, gender, ethnicity, employment status) and questions related to their pain experience [21] and perception and beliefs [22]. We assigned a 4-digit code to all consenting participants and followed established institutional guidelines to ensure confidentiality of their data. They also received workshop details via email and were offered support with joining the web-based workshop, if needed. Two researchers (SMA and SNvdV) facilitated the workshops and presented the ground rules for the session at the start of the workshop (eg, providing a safe space for sharing opposing opinions, keeping discussions private within the group). Representatives from uMotif Limited (BJ and SMA), our technology partner, developed and presented the mock screens for feedback but they were not involved in other aspects of the data collection or in the data analysis.

### Cultural Similarities and Differences in Pain Experience and Its Reporting

The topic guide for the focus group discussions on pain experience and reporting and its relationship with culture (objective 1) was informed by the literature [21,23-25] and included topics such as pain experience, pain perception, pain report and communication, and pain assessment. Focus group discussions were audio-recorded and transcribed verbatim. Once the transcriptions were ready, we anonymized the transcripts and destroyed the audio recordings.

### User Requirements for Digital Pain Self-report Tools

**Figure 1:**
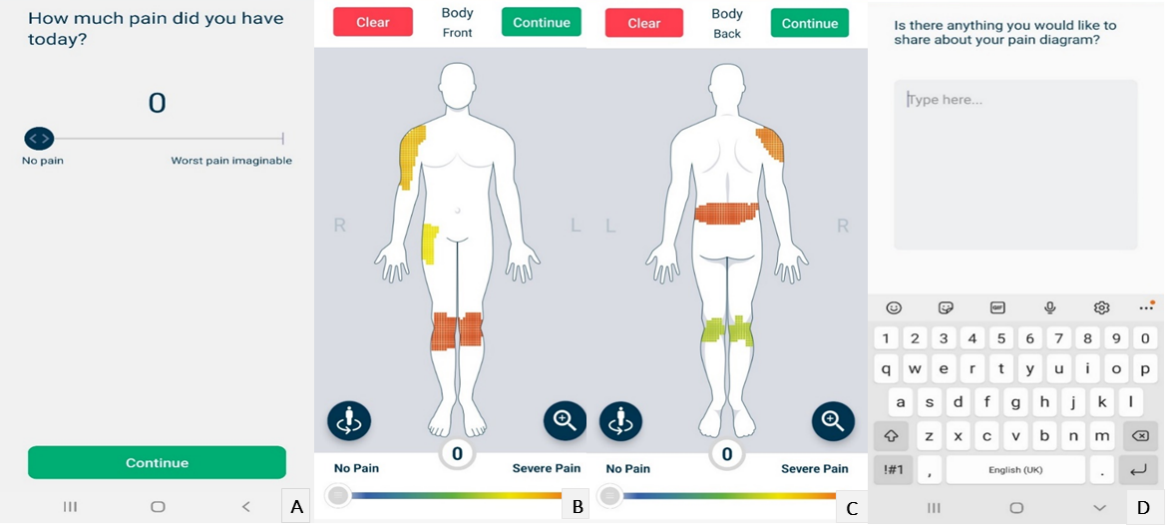
Screenshots of the Manchester Digital Pain Manikin app (developed by uMotif Limited, copyright University of Manchester and uMotif, 2020), which we used as an example of a digital pain self-report tool. A. Numeric rating scale for overall pain intensity; B. Front view of the body manikin with pain drawing; C. Back view of the body manikin with pain drawing; D. Pain diary.

To prompt discussions on important aspects of pain self-reporting (objective 2), we demonstrated the Manchester Digital Pain Manikin app [26]—developed by uMotif Limited—as an example of a digital pain self-report tool (see Figure 1). People can use the app to report overall pain intensity on a numeric rating scale from 0 to 10, location-specific pain intensity on a 2D gender-neutral body manikin, and a free text pain diary to elaborate on the manikin drawing. After the demonstration, focus group participants were split into 2 smaller breakout groups to discuss user requirements, including what they would want to report about their pain (ie, pain aspects) and how (ie, app features) and why they considered these aspects and features important (see Table 1). Digital pain self-report tools can have multiple purposes (including supporting self-management, guiding clinical decisions, collecting data for research), and we did not specify any particular purpose at the start of these discussions. The facilitators recorded the breakout groups’ responses in a shared Google doc.

**Table 1:** Questions to guide breakout group discussions on user requirements for the pain self-report tool.

Question type	Question on user requirement
What (Q1)	What pain aspect or app feature would be important for you to report?
Why (Q2)	Why is that aspect or feature important?
How (Q3)	Do you feel the aspect or feature is currently available in the Manchester Digital Pain Manikin app?

### Data Analysis and Synthesis

#### Cultural Similarities and Differences in Pain Experience and Its Reporting

To analyze the transcripts of the focus group discussions (objective 1), we utilized an interpretive analysis approach, also referred to as hermeneutic phenomenology [27]. This approach has been used in previous studies to understand the lived experience of pain [28]. Two researchers (SMA and RRL) reviewed the transcripts line-by-line independently to immerse themselves in the data; both had experience of qualitative data collection and analyses in the fields of public health and health psychology, respectively. One researcher (SMA) assigned codes to all relevant statements to find patterns, linked them across transcripts, and discussed these in the context of the participants’ cultural background with the other researcher (RRL). Both researchers used their own cultural background to interpret textual data, codes, and themes, which emerged from the data. SMA recorded all the emerging codes in a codebook alongside illustrative quotes, iteratively refining the codebook after reviewing each transcript and discussing them with RRL. Once the codebook was finalized, SMA reapplied it to all the transcripts and drew themes to ensure consistency. Under each theme, we first synthesized similarities in people’s pain experience across ethnic groups and then highlighted differences in their viewpoints that may be linked to their ethnic backgrounds. We managed all qualitative data (ie, codebook, illustrative quotes) by using Microsoft Excel.

#### User Requirements for Digital Pain Self-report Tools

User requirements for pain self-report tools related to important pain aspects and app features (objective 2) were thematically synthesized by 2 researchers (SMA and SNvdV) to identify similarities and differences between ethnic groups. We then invited participants from across ethnicity-specific workshops to attend another web-based workshop. People could express their interest via email and were offered a place on first come, first served basis, while ensuring a balanced representation across ethnic groups. The aim of the workshop was to check for accuracy of our findings and whether these resonated with participants’ experiences and preferences (ie, member-checking exercise). For this, we asked participants for feedback on our synthesis of the user requirements as well as on mock-ups for the Manchester Digital Pain Manikin app to illustrate how some of the identified key requirements for pain self-report tool could be translated into app functionalities.

## Results

### Participants’ Characteristics

In total, 23 adults (14 females, 61%) took part across the workshops. Table 2 shows that the majority of the participants (13/23, 56%) were aged 45 years and older, did not have English as their native language (12/23, 52%), and had experienced pain for 4 years or more (15/23, 65%). Regarding participants’ pain perception and beliefs, all thought that pain intensity varied but was always present, and 14 (61%) participants felt that they did not know enough about their pain.

**Table 2:** Characteristics of the study participants (N=23).

Characteristics, response categories		Values, n (%)
**Age (years)**		
	25-34	5 (22)
	35-44	5 (22)
	45-54	4 (17)
	55-64	7 (30)
	65+	2 (9)
**Gender**		
	Male	9 (39)
	Female	14 (61)
**Ethnicity**		
	South Asian^a^	10 (45)
	Black African	6 (27)
	White British	7 (32)
**Employed**		
	Yes	10 (45)
	No	13 (55)
**Is English your native language?**		
	Yes	11 (48)
	No	12 (52)
**How long have you been experiencing pain? (years)**		
	≤3	8 (35)
	4-10	6 (26)
	>10	9 (39)
**My pain varies in intensity but is always present**		
	Agree	23 (100)
	Disagree	0 (0)
**I do not know enough about my pain**		
	Agree	14 (61)
	Disagree	9 (39)
**If I am in pain, it is my own fault**		
	Agree	5 (22)
	Disagree	18 (78)

^a^Included people from Pakistani and Bangladeshi backgrounds.

### Cultural Similarities and Differences in Pain Experience and Pain Reporting

Participants across all ethnic groups indicated that their culture influenced how they perceived pain (eg, what causes pain), how they managed it (eg, whether to take medication), and how they communicated about their pain and with whom. Four main themes emerged from our interpretive analysis, namely, perceived causes of pain, approaches and attitudes to self-treatment and management, frustration and embarrassment when communicating about pain with others, and lack of experience with formal pain assessment tools. Below, we describe each theme in more detail alongside selected illustrative quotes, with additional quotes supplied in Multimedia Appendix 3.

#### Theme 1: Perceived Causes of Pain

Most participants described their pain experience as agonizing and explained how it was to live with pain, what caused their pain in their perception, and what impact it had on them. Across ethnicities, participants described their pain in similar ways, including that they were always in pain but that it fluctuated. They referred to good days or bad days when pain was less or more, respectively. Female participants talked about gender norms such as caring responsibilities and domestic chores as an inevitable cause of their pain.

…Females do all the domestic chores and with time and age it's bound to happen. These things are supposed to kick in and women do complain…so complaining about pain is just the norm for women I think.

South Asian female

Only participants from South Asian background perceived food type to be a cause of their pain.

…Food that has a lot of spice, perhaps has a lot of oil, and you use ghee based substances, which can cause a greater reaction in my opinion.

South Asian male

South Asian and White British participants also perceived weather conditions to be a potential cause.

…It could be the weather here, because when I go to…like I've been to Spain, I've been to Pakistan, Dubai, it's very hot there. And you don't feel much pain there.

South Asian female

…because winter is the time when it really gets more and more kind of affected.

White British male

Across all ethnic groups, the negative effects of pain on mental health were consistently mentioned and the participants expressed their mental state as brain fog, confused, stressed, dementia-like, trauma, and bad mood.

…it's just that as it [pain] progresses it was affecting my memory as well.

Black African female

Similarly, participants across all ethnic groups mentioned how their pain negatively affected their relationship with family members.

#### Theme 2: Approaches and Attitudes to Self-treatment and Management

Participants across all ethnic groups expressed their dissatisfaction with the treatment they were currently receiving. They also described how they relied on self-management practices and on pacing themselves to manage their painful condition better. Thinking about the diagnosis of their painful conditions, some participants said pain was an unexpected diagnosis for them, while others expressed frustration about delays in having their condition diagnosed as such.

…I had to run to my GP on many occasions…to explain that I'm suffering with this pain and I want to get to the bottom of what it is…and the doctor said to me oh, you're still young. You're still in your 20s. You can't have this [painful condition].

South Asian female

Participants also expressed concerns about treatment effectiveness and how they were given different treatments and but remained unable to manage their pain effectively. Participants discussed how they developed the practice of self-medication.

…I now self-medicate myself according to the level of pain that I’ve actually got.

White British male

For managing pain, a participant described medication practice with a cultural viewpoint.

…We tend to tolerate it perhaps in a different way, and adjust really the cultural issue of not using medications or tablets as, almost like sweets. So we tend to only use medication where it's absolutely necessary.

Black African male

#### Theme 3: Frustration and Embarrassment in Communicating About Pain With Others

Communicating about pain with friends, family members, and health care professionals was described as challenging across all ethnic groups. One of the participants described how communicating pain history during consultations was particularly difficult.

…And having to do some consultation, I get irritated because asking me to check my joints…how would I know what to do? How do I… I can't tell my progress in a week, in a month. I really can't unless I keep a diary of what's going on….

Black African female

South Asian participants, particularly women, shared feeling embarrassed when talking about their pain.

….I think it's the way you’re brought up…some people find it embarrassing, that shouldn't be discussed with the rest of the family.

South Asian female

Male participants also described how the image of masculinity in their culture and the need for preserving their self-image hindered them to talk about their pain, which led them to developing a negative reporting behavior.

…men are more resistant to expressing their medical conditions because they are so much…stronger and it's not supposed to be…like a man to complain about anything.

Southeast Asian male

A White British participant shared a similar perspective on self-image but less directly linked to his cultural background compared to South Asian participants.

…But for me, talking to others is about managing my own self-image. Because…people see me in a particular way. And the fact that I’m unable to do certain things….reduces me in some way, in my own mind, to some extent. And so I tend not to talk.

White British male

Black African participants mentioned pain was perceived as a disability in their culture, thereby reinforcing their negative reporting behavior.

…Disability is not something that is seen as something to talk about in our culture. You just want to hide things and just behave as if everything is okay.

Black African female

With hiding disability being the norm, it also limited them to optimally manage their pain.

…So that's another problem. You're not sure if using the aid will make you better. But then you don't want to use it because of the attention it creates as well. So all this contributes to the mental struggle.

Black African female

Communicating pain to health care professionals was found to be equally challenging.

…Just in general, I find it very hard to communicate with medical professionals where the pain is, what it feels like, the very fact that it’s even real.

White British female

A Black African participant expressed how it could be more beneficial to speak to a health care professional with a similar cultural background.

…But like others are saying, honestly, if [my doctor] came from the same background as mine…he was African…I think it would have been better to explain how the pain was going. Because we've got the actual words to actually explain how the pain is like.

Black African female

With regard to describing pain in culturally appropriate and understandable language or terms, the following 2 contrasting opinions were noted.

…I was lucky. My GP is of Asian background but he lived in Africa. So it was more like a fatherly conversation kind of thing. So that helped as well because he understood where I was coming from.

Black African female

…I went to my GP a while back, the GP was Gujarati [South Asian] and I just didn't feel comfortable disclosing my issues to him.

South Asian male

#### Theme 4: Lack of Experience With Formal Pain Assessment Tools

Few participants had experience of completing pain self-assessment tools as part of their care, and those who had completed were unhappy because they thought pain reporting methods did not capture their pain situation comprehensively.

…there's a picture of a person and you have to put a cross on the places where you've got pain. But…that just tells them there's pain in that area. It doesn't give them a good indication of how much pain, whether it's worse in certain areas than others.

South Asian female

…So being told to grade the pain to a physician is very, very difficult for me to do.

Black African female

…The GP was instantly like the others, just saying, is it every day, on a scale of 1 to 10 what is it? And you just feel so rushed that you don’t get a chance to explain that no, it’s not every day but some days it’s bad at a certain time in the day.

White British female

Some participants who had experience of reporting pain using the Manchester Digital Pain Manikin app in our feasibility study (Ali SM et al, unpublished data, January 2023) described their experience as follows:

…I felt like describing my pain to someone. I thought someone's listening to me, someone's understanding it.

South Asian female

…something like this [a smartphone app] would be very ideal in the context that it would be very confidential. I would have the opportunity to input area of my pain to get better advice.

South Asian male

### User Requirements for Digital Pain Self-report Tools

In total, 21 user requirements across 4 categories emerged from the synthesis of participants’ views on what pain aspects and app features were important (see Table 3). Nine requirements were consistent across ethnic groups, while 12 were only mentioned during one of the ethnicity-specific workshops. Below, we summarize per category similarities and differences in requirements between ethnic groups and how differences were discussed during the member-checking workshop; we did not discuss similarities during this workshop.

**Table 3:** User requirements for digital pain self-report tools.

Pain aspects/app features		Ethnicity-specific workshops^a^			Member-checking workshop^b^	Remarks^c^
		South Asian	Black African	White British		
**Location-specific pain aspects**						
	Pain quality (eg, stabbing, throbbing)	Yes^d^	Yes	Yes	Not discussed	Helps to characterize the medical condition
	Location-specific pain intensity	Yes	Yes	Yes	Not discussed	Pain intensity may differ by body location
	Pain radiation	Yes	No	Yes	Agreed^e^	Shows where the pain spreads to
	Pain layers or depth^f^	Yes	Yes	No	Agreed	Helps to differentiate the problem and adds precision; tells which part of the musculoskeletal system (bone, muscle, or joint) is affected
	New pain	Yes	Yes	Yes	Not discussed	Helps to identify when pain started in a certain location and to track how it developed
	Pain timing/duration	No	Yes	Yes	Agreed	Helps to distinguish continuous from intermittent from constantly varying pain; keep track of how long a location has been painful (or pain-free).
**Non–location-specific pain aspects**						
	Pain causes and aggravating factors (ie, factors that cause or increase)	Yes	Yes	Yes	Not discussed	Helps to understand how to manage pain
	Pain impact (ie, interference with other activities or consequences)	Yes	Yes	Yes	Not discussed	Provides insights into what other conditions you are developing because of your pain
	Pain management strategies	Yes	Yes	Yes	Not discussed	Helps to keep track of how you are managing your pain (eg, medication, swimming)
	Semistructured diary field (with headings as suggestions for what to record in this field)^f^	No	No	Yes	Agreed	Allows recording of additional relevant information (eg, diet, physical activity, level of medication)
	Reasons for not reporting pain	No	Yes	No	Not agreed	Enables capturing of bad days when unable to complete a report or pain-free days when there was nothing to report
**App features: Feedback and output**						
	Feedback of previous pain reports^f^	Yes	Yes	Yes	Not discussed	Helps to see relationship between pain levels and for example, pain management strategies
	Pain management guidance based on pain reports	No	Yes	Yes	Agreed	Supports pain management
**App features: Look and feel**						
	Available for any digital device	Yes	Yes	Yes	Not discussed	Increased accessibility
	Flexible reporting frequency	No	Yes	Yes	Agreed	Allows reporting whenever pain changes over the course of the day
	Multiple languages	Yes	Yes	No	Not agreed	Increased accessibility
	Intuitive color scheme linked to pain intensity scores	Yes	Yes	Yes	Not discussed	Enhances interpretation of pain reports
	Manikin zoom-in function (by finger pinch)	No	No	Yes	Not agreed	Enables easier reporting of pain location
	Manikin body sides (minimum front, back, lateral sides)	Yes	No	No	Agreed	Enables more accurate reporting of pain location
	Manikin detail	Yes	No	Yes	Agreed	Increased relevance to user; more life-like
	Manikin personalization (eg, gender-specific)^f^	No	Yes	Yes	Agreed	Increased relevance to user; more life-like

^a^Requirements that were mentioned during the ethnicity-specific workshop are represented as yes and those that were not mentioned as no.

^b^Consistently reported requirements across all ethnicity-specific workshops were not discussed during the member-checking workshop and are therefore shown as not discussed. For requirements that were discussed, agreement across participants is represented as agreed and lack of clear agreement as not agreed.

^c^Summary of the illustrative participant comments noted during breakout groups (in ethnicity-specific workshops) to clarify why people considered certain pain aspects and app features important.

^d^Yes means a pain aspect or app feature was mentioned during a particular ethnicity-specific workshop.

^e^Agreed means participants agreed on its importance during the member-checking workshop.

^f^Requirement presented as a mock-up screen for the Manchester Digital Pain Manikin app to gather further thoughts on how the requirement could be translated into a functionality.

### Location-Specific Pain Aspects

During 2 ethnicity-specific workshops (South Asian and Black African) and the member-checking workshop, participants identified pain layers (eg, skin, muscle, bone) as an important aspect. However, when we showed mock-ups of how this could be implemented in the Manchester Digital Pain Manikin, participants reported that this might overcomplicate pain reporting, suggesting that translating this requirement into a functionality may not be straightforward. When discussing pain radiation and pain duration as aspects during the member-checking workshop, participants agreed these were relevant for the digital pain self-report tool.

### Non–Location-Specific Pain Aspects

All participants mentioned that they would be motivated to regularly self-report their pain if this would enable them to manage their pain better. Across workshops, participants described reporting of pain causes or aggravating factors crucial in this context. However, we found during the focus groups that the type of perceived pain causes varied across groups. Only White British participants suggested a semistructured diary field to capture information about diet, mood, physical activity, and level of medication, which participants from the other 2 ethnic groups appreciated during the member-checking workshop when shown mock-up screens for this functionality. They additionally suggested that such a diary field could be linked to a specific pain location to enable location-reporting of factors associated with pain (eg, perceived pain causes).

### Feedback and Output

Participants wanted summaries of their pain reports, which in their view would enable them to track pain fluctuations in relation to changes in management and coping strategies. Participants confirmed this requirement during the member-checking workshop after seeing mock-ups of the pain summary reports while also sharing additional thoughts on how best to summarize the changes in pain, medication use, and coping strategies. Black African and White British participants also suggested that personalized data-informed messages could, for example, encourage people to refrain from undertaking activities that seemed to aggravate their pain to which South Asians also agreed during the member checking.

### Look and Feel

Participants considered showing the lateral sides of the manikin (instead of just front and back) and manikin personalization (eg, option to choose a male or female manikin) important for their pain self-reporting. Mock-up screens showing manikin personalization options for gender and body shape were shared for participants’ feedback. They had mixed opinions about gender, while expressing a shared but negative opinion about the presented personalization options for body shape, as they felt it might offend some people or make them overly conscious of their bodies. South Asian participants thought that translating instructions into their native language would reduce barriers to pain self-reporting. Similarly, Black Africans suggested that the use of culturally appropriate pain terminologies would be beneficial. For example, the term “pain quality” may only make sense to South Asians if accompanied by examples and visualizations of types of pain quality (eg, icons representing tingling, stabbing). Lastly, participants commented on how the pain intensity scale and color scheme could be described more meaningfully (eg, by describing pain intensity).

## Discussion

### Summary of Findings

We conducted 3 web-based focus groups followed by a user requirement exercise with people from different ethnic backgrounds living with a chronic pain condition. We found many similarities in how the participants described their experience of living with pain; how pain management is still suboptimal; and how it is challenging to communicate about pain with their friends, family members, and health care professionals. People from non-White ethnic backgrounds had different beliefs and perceptions on pain compared to those from White backgrounds, which resulted in internalizing stigma and developing a negative attitude toward medication and pain reporting. Despite these differences, participants across ethnic backgrounds agreed on which aspects of pain reporting were important to self-report, such as pain quality, pain causes, feedback of previous pain reports, and availability of a digital device for pain management. However, we found differences in requirements related to language (eg, translated in-app instructions, culturally appropriate pain terminologies) and that people did not always agree on how best to translate requirements into reporting functionality (eg, pain layers/depth). Addressing these differences when developing digital pain self-report tools will enhance their cross-cultural acceptability and contribute to more equitable pain management and outcomes by reducing pain reporting barriers across ethnic groups.

### Relation to Other Studies

We found that gender stereotypes and associated stigma, which may vary across cultures, influenced people’s pain experience and reporting behavior negatively. For example, Black African female participants in our study said that pain is viewed as a disability, leading to negative disclosure behavior (ie, people are less likely to report their pain). This aligns with findings from a review by Bakhshaie et al [9] in 2022 who suggested that stigma internalization (eg, when somebody links their disability to their personality) in Black individuals results from the interplay between interpersonal, community, and societal factors, which in turn is related to discrimination and societal injustice [9]. Similarly, South Asians indicated that pain among women is considered inevitable because of women’s household responsibilities. Owing to the conventional gender roles, men may be less willing to report pain and more willing to endure it [29]. This finding is in line with those reported in other studies [30,31] that specific expectations evoked by gender, ethnicity, nationality, or religion may further complicate pain experience.

We found that there was a general criticism among participants about single-rating scales and other existing tools. One issue they highlighted was that they found those tools too simplistic for their complicated pain situation. The identification of different pain aspects, for example, intensity, quality, frequency, duration, and their temporal aspects; pain causes; and impacts are consistent with recommended core outcome measures for chronic pain [32]. In addition, assessment tools for pain self-reporting may affect the patient-provider encounter and lead to unintended results if they are used with a culturally and linguistically diverse population [33]. Further, a cross-cultural validation study found differences between ethnic groups for pain quality descriptors such as aching, gnawing, and throbbing, possibly because of cultural and linguistic differences [34]. This may partly explain why we found general support for visual methods of pain assessment (such as pain manikins) among people across ethnicities, assuming they allow tailoring to cultural reporting needs [35] such as the culturally perceived pain causes and use of acceptable pain terminologies suggested by the participants in our study.

### Limitations of This Study

One limitation of our study was that the samples for each of the ethnicity-specific workshops were relatively small and may not have reflected the wide range of cultural diversity within a specific ethnic group. For example, the Pakistani culture comprises numerous ethnic groups such as Punjabis, Kashmiris, Sindhis, and Muhajirs. Therefore, specific pain belief and pain self-reporting needs within ethnic subgroups and examining to what extent these beliefs and needs are common in such subgroups across countries (eg, Punjabis living in Pakistan, India, and the United Kingdom) is an area of future research.

Another limitation was that only people who spoke and understood English and who had access to a digital device and the internet could take part in the workshops. This may have further reduced the diversity of our sample. For example, people with a disability or those who are older are less likely to use the internet [36]. Similarly, although South Asians are more likely to experience chronic pain [5], not all may be sufficiently proficient in English to participate in group discussions, thereby limiting the generalizability of our findings. Future studies could therefore consider conducting in-person interviews or focus group discussions in people’s own language (eg, Urdu) at a convenient place (eg, a community center). In addition, as these people are more likely to represent less affluent groups, engaging with them would help us examine the intersectional considerations (related to ethnicity; eg, income level, occupation type, education level) within a specific ethnic group.

### Implications for Developing Cross-culturally Acceptable Digital Pain Self-report Tools

People across ethnic groups mostly agreed on what were relevant and important aspects of pain, which included pain causes. However, differences in perceived pain causes between ethnicities, such as food, weather, and gender norms, should be acknowledged to facilitate culturally relevant pain self-reporting that supports people with self-managing their pain. Similarly, digital pain self-report tools such as smartphone-based pain manikins showed potential in overcoming challenges of communicating pain with health care professionals, especially for people from non-White ethnic backgrounds, which suggests that pain drawings may have clinical utility [37]. However, this requires cultural (eg, culturally appropriate pain terminologies) and linguistic (eg, translated instructions in users’ native language or use of audio/video instead of text) compatibility across a wide range of ethnic backgrounds.

In addition to these features, digital tools incorporating a pain manikin should offer the option of personalizing the body shape [17]. However, our experience from the member-checking workshop showed that it is not straightforward to translate user requirements related to manikin personalization into app functionalities that meet people’s expectations. Further, adding more functionalities to increase cultural and gender appropriateness needs balancing against increasing the complexity of using the pain self-report tool as intended to avoid creating barriers for other potentially disadvantaged groups (eg, those with lower digital literacy levels or limited manual dexterity). Lastly, offering personalization options may affect the measurement properties of digital manikins and how we interpret manikin drawings and the data derived from them. Developers of digital manikins and researchers should further explore how best to address the need for manikin personalization and its impact on data collection, analysis, and interpretation.

We need innovative user-centered prioritization techniques to facilitate the development of equitable digital pain and other health assessment tools. Currently, methods for prioritizing requirements, which emerged from an increased need to involve stakeholders in developing software and information systems [38], are commonly based on majority votes, for example, the Top10, cumulative voting, and numerical assignment [39]. However, in our study, we found some user requirements that were only relevant to a specific minority group, and existing prioritization techniques insufficiently encourage developers to appreciate these.

### Conclusion

Exploring the views of people from different ethnic backgrounds generated new insights into their pain experiences and challenges in communicating their pain. There were cultural differences in perceived causes of pain, self-management strategies, and their reporting behavior because of gender norms and the stigma associated with pain. Moreover, there were differences in language requirements. Acknowledging and addressing these differences is important for the development of cross-culturally acceptable digital pain self-report tools, which in turn will contribute to reducing inequities in pain treatment and outcomes.

## Appendix

Multimedia Appendix 1 Study flyer.

Multimedia Appendix 2 Topic guide.

Multimedia Appendix 3 Additional illustrative quotes.
