# Supplementary material for: Exploring the Cross-cultural Acceptability of Digital Tools for Pain Self-reporting: Qualitative Study
Source: JMIR Hum Factors. 2023 Feb 8;10:e42177. doi: 10.2196/42177 (PMC9947768; doi:10.2196/42177)
Supplement: Multimedia Appendix 1 [file humanfactors_v10i1e42177_app1.pdf]

THE MANCHESTER DIGITAL PAIN MANIKIN

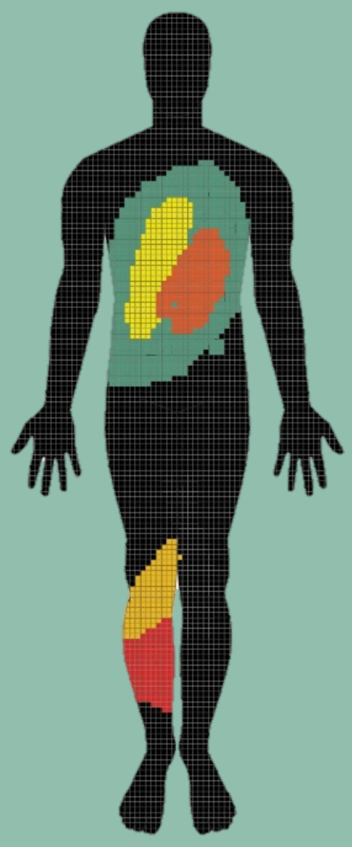

DO YOU HAVE ARTHRITIS OR ANY OTHER LONG-TERM MUSCULOSKELETAL PAIN CONDITION?

ARE YOU INTERESTED IN IMPROVING THE WAY HOW LONG-TERM PAIN IS MEASURED?

×××××××××××××××

We are looking for people with arthritis or any other long-term musculoskeletal pain condition to attend a 2 hour workshop. The workshop will help us understand how you talk about your pain with others, and how the Manchester Digital Pain Manikin could support you with that

ARE YOU?

- AN ADULT MALE OR FEMALE (AGED 18 OR ABOVE)
- AFFECTED BY ARTHRITIS OR ANY OTHER LONG-TERM MUSCULOSKELETAL PAIN CONDITION
- ABLE TO SPEAK, READ AND UNDERSTAND ENGLISH
- A WHITE BRITISH BY ETHNIC BACKGROUND
- A SMARTPHONE USER

THEN

JOIN US!

SEPTEMBER 27, 2021 | 10 AM - 12 PM

VIA ZOOM

(LINK WILL BE SHARED LATER)

For more information, please contact:
Mustafa Ali
E-mail: painmanikin@manchester.ac.uk;
Contact number: 07459073783

MANCHESTER 1824
The University of Manchester

You will be compensated for your time

The Manchester Digital Pain Manikin Study Flyer
Version 1.1., 07/12/2020
IRAS ID: 293246
