# Supplementary material for: Exploring the Cross-cultural Acceptability of Digital Tools for Pain Self-reporting: Qualitative Study
Source: JMIR Hum Factors. 2023 Feb 8;10:e42177. doi: 10.2196/42177 (PMC9947768; doi:10.2196/42177)
Supplement: Multimedia Appendix 2 [file humanfactors_v10i1e42177_app2.docx]

**Multimedia Appendix 2**

**TOPIC GUIDE**

Can you please explain your pain experience (historically)?

- How long you have been experiencing pain?
- How you have been managing it? For example, by dietary supplements, resting and etc.

In your opinion, what causes your pain? Are there any things that influence your pain *[concerns pain perception]*

- Explore relevance of medical condition
- Explore the influence of ethnicity and culture (family dynamics, gender norms) and other demographic factors, such as age, gender, occupation on how people experience and manage their pain.
- Assess whether demographic factors outweigh medical factors

What are the situations when you decide to talk about your pain? [concerns pain reporting behaviour]

- With whom you discuss your pain and why? With friends, family and doctor?
- When do you think it is essential to report your pain to a doctor?
- What are those situations in which people prefer talking to friends/family rather than their doctor, and vice versa?

Can you tell me about your experience of talking to health care professionals about your pain?

- What difficulties or challenges you particularly face for seeking healthcare services?
- What is the influence of doctor’s ethnicity on your choice of pain reporting behaviour?
- Explore language barriers. Are there common words or terms between patient-provider?
- Explore one’s ability to communicate his/her pain experience, even if there is no language barrier

What is your experience of self-reporting your pain using questionnaires and/or digital tools [show example questionnaires or digital tools as prompts]?

- Did you have any difficulty in completing questionnaire? Elaborate each
- Which method (digital or paper-based) is acceptable for you to report your pain?
- What can facilitate your pain reporting? Explain each factor. For example, length or wording of the questionnaire,

Do you want to say anything else regarding your pain experience and reporting it?
