# Supplementary material for: Exploring the Cross-cultural Acceptability of Digital Tools for Pain Self-reporting: Qualitative Study
Source: JMIR Hum Factors. 2023 Feb 8;10:e42177. doi: 10.2196/42177 (PMC9947768; doi:10.2196/42177)
Supplement: Multimedia Appendix 3 [file humanfactors_v10i1e42177_app3.docx]

**Multimedia Appendix 3**

**Additional illustrative quotes**

| **Themes** | **Additional illustrative quotes** |
| --- | --- |
| Pain, its causes and effects | *“I would just say living in pain perpetually is a very difficult condition” [black African woman]* |
|  | *“Even one single finger touch, you feel like you're in severe pain, they stab like a knife in the back” [southeast Asian woman]* |
|  | *“I feel it sort of all my muscles hurt all of the time, they just hurt more or less depending on what I’m doing” [white British woman]* |
|  | *“when you have something like fibromyalgia ... very often … new pain gets dismissed as part of … your symptoms and nobody ever kind of looks at that pain as a whole and tries to help”* [white British woman] |
|  | *“…it's like you're not sure if it's you, it's your thinking that is manifesting the pain or you're actually in pain”* [black African woman] |
|  | *“But yeah, pacing is very difficult when you have a job and small children and lots of other life pressures as well”* [white British woman] |
|  | *“So I’m quite lucky… I guess that my mum also has fibromyalgia, so she has a complete understanding. So the two of us are as supportive to each other as possible”* [white British woman] |
| Pain treatment and management | *“……I think because I’ve had so much pain in so many different places and for so long, my way of coping with it is really to try to ignore it. I always do my best to do that*” [white British woman] |
|  | *“I think it's probably a lot to do with cultural upbringing, having the tolerance to pain”* [black African man] |
|  | *“I needed...someone to tell me that I wasn’t going to make it worse by doing certain things”* [white British male] |
|  | *“When it's bad you're just on the bed, constantly. You're very stiff, you can't do daily stuff. But when you're on the move you can do much. But you're scared in case you might pull a muscle, you might make it worse. So you do get worried when you're doing some activities”* [southeast Asian woman] |
| Communicating pain with others | *“when you are talking to other people, especially if you're getting quite passionate, I end up just smacking my knees, and it's the numb, basically. You are showing your strength, you're saying that it doesn't affect you. It's not really a big problem because other men laugh it off if you talk about the pain”* [Southeast Asian male] |
|  | *“there’s almost like a sense of shame when you suffer from chronic pain that you can’t manage it. Because there’s always people around who claim that they’re in absolute agony and yet they’re still able to…”* [white British female] |
|  | *“Whereas with friends, I can just be more myself and I know that I won’t be judged in that way”* [white British male] |
|  | *“We seem to joke about it, we don't take it serious enough. But really this is a serious thing, what's happening”* [Southeast Asian male] |
| Experience with pain assessment | *“I just found it a bit useless...They take the paper and then they just file it. They don't do anything with it, and that's the problem when you give it to them”* [southeast Asian male] |
|  | *“[a smartphone app] seems to be a really good idea. Because you forget what’s going on and what was…when you had a flare-up, you don’t know how long it lasted because once it’s gone, you’re so relieved that you can’t remember it being a score 10 or a score nine”* [white British female] |
